# Bioinformatics Analysis Reveals Diagnostic Markers and Vital Pathways Involved in Acute Coronary Syndrome

**DOI:** 10.1155/2020/3162581

**Published:** 2020-11-06

**Authors:** Mingshuang Li, Conglin Ren, Chenxia Wu, Xinyao Li, Xinyi Li, Wei Mao

**Affiliations:** ^1^Department of Cardiology, The First Affiliated Hospital of Zhejiang Chinese Medical University, Hangzhou, Zhejiang 310002, China; ^2^The Third Clinical Medical College of Zhejiang Chinese Medical University, Hangzhou, Zhejiang 310051, China; ^3^Department of Cardiology, Zhejiang Hospital, Hangzhou, Zhejiang 310013, China

## Abstract

**Background:**

Acute coronary syndrome (ACS) has a high incidence and mortality rate. Early detection and intervention would provide clinical benefits. This study aimed to reveal hub genes, transcription factors (TFs), and microRNAs (miRNAs) that affect plaque stability and provide the possibility for the early diagnosis and treatment of ACS.

**Methods:**

We obtained gene expression matrix GSE19339 for ACS patients and healthy subjects from public database. The differentially expressed genes (DEGs) were screened using Limma package in R software. The biological functions of DEGs were shown by Gene Ontology (GO), Kyoto Encyclopedia of Genes and Genomes (KEGG), and Gene Set Enrichment Analysis (GSEA). Protein-protein interaction (PPI) network was mapped in Cytoscape, followed by screening of hub genes based on the Molecular Complex Detection (MCODE) plug-in. Functional Enrichment analysis tool (FunRich) and Database for Annotation, Visualization and Integrated Discovery (DAVID) were used to predict miRNAs and TFs, respectively. Finally, GSE60993 expression matrix was chosen to plot receiver operating characteristic (ROC) curves with the aim of further assessing the reliability of our findings.

**Results:**

We obtained 176 DEGs and further identified 16 hub genes by MCODE. The results of functional enrichment analysis showed that DEGs mediated inflammatory response and immune-related pathways. Among the predicted miRNAs, hsa-miR-4770, hsa-miR-5195, and hsa-miR-6088 all possessed two target genes, which might be closely related to the development of ACS. Moreover, we identified 11 TFs regulating hub gene transcriptional processes. Finally, ROC curves confirmed three genes with high confidence (area under the curve > 0.9), including VEGFA, SPP1, and VCAM1.

**Conclusion:**

This study suggests that three genes (VEGFA, SPP1, and VCAM1) were involved in the molecular mechanisms of ACS pathogenesis and could serve as biomarkers of disease progression.

## 1. Introduction

Acute coronary syndrome (ACS) is an acute cardiovascular event with rupture or invasion of coronary atherosclerotic plaque as pathological basis, followed by the formation of complete or incomplete occlusive thrombus, which has high morbidity and mortality in developed and developing countries [1, 2]. The fissure of atheromatous plaque causes thrombotic reaction and coronary artery blood flow obstruction, which leads to downstream myocardial ischemic damage. Abnormalities in function and structure of the coronary microcirculation also lead to the development of ACS [3, 4]. Risk factors for ACS include, but are not limited to, aging, unhealthy lifestyles, obesity, diabetes, and high blood pressure. Moreover, it is worth noting that ACS occurs more often in males and those with a family history of the disease [5]. Maintaining plaque stability and preventing plaque rupture are paramount measures to prevent ACS. Percutaneous coronary intervention (PCI) and timely restoration of perfusion can reduce irreversible myocardial injury and improve the prognosis of ACS [6]. Despite aggressive treatment, ACS may still be associated with a number of potential complications that can reduce a patient's quality of life and even affect survival time. Therefore, seeking reliable biomarkers is particularly important for the early diagnosis and medical intervention of ACS.

The main factor of plaque formation is elevated cholesterol levels caused by an imbalance between influx and efflux, resulting in abnormal accumulation of lipids in the lining [7]. The pathological change associated with early atherosclerosis is the formation of macrophage-derived foam cells [8]. In this process, circulating monocytes enter the subintima and differentiate into macrophages, which take up oxidatively modified lipoproteins via scavenger receptor class A (SRA) and CD36 [9]. On the other hand, lipoproteins transform macrophages into lipid-rich foam cells, which is thought to be a key step in the development of atherosclerosis and a major contributor to chronic inflammation [10].

Numerous researches have been made to develop clinical detection indicators. Creatine kinase-MB, cardiac myoglobin, and cardiac troponin *I* and *T* have been widely applied in clinical diagnosis of acute myocardial infarction [11]. With the development of whole genome sequencing and the boom in bioinformatics research, it is possible to find new and more sensitive biomarkers for ACS. It is worth noting, however, that some previous studies yielded controversial or even contrary results, which may be attributed to different sample selection, batch effects between groups, and differences in operational protocols.

In this study, we compared gene expression differences between patients with ACS and healthy individuals, with the aim of uncovering clinical biomarkers and analyzing their biological functions, which could help to elucidate the pathogenesis of ACS and, consequently, explore potential therapeutic strategies.

## 2. Materials and Methods

### 2.1. Data Acquisition

The microarray expression dataset GSE19339 and its annotation file GPL570 were retrieved from Gene Expression Omnibus (GEO) database. A total of eight samples were analyzed in this study, four of which were leukocytes from thrombus of ACS patients and the other four were peripheral blood leukocytes from healthy controls.

### 2.2. Data Processing

We used RStudio software to process the raw expression matrix. In short, probe IDs were converted to gene symbols using platform annotation file and well-annotated probes were retained. For missing values in the dataset, we used the KNN method of the impute R package for auto-fill. If one gene was detected by multiple probes, its average expression value was used for subsequent analysis.

### 2.3. Differential Expression Analysis

The differentially expressed genes (DEGs) between ACS samples and healthy controls were analyzed by Limma package [12]. The screening criteria were as follows: |log2 fold change (FC)| > 2 as well as adjusted *p* value < 0.05. The visualization of DEGs was presented through volcano plot and heat map using ggplot2 and pheatmap R packages, respectively.

### 2.4. Function Enrichment Analysis

Gene Ontology (GO) and Kyoto Encyclopedia of Genes and Genomes (KEGG) pathway analysis were carried out based on clusterProfiler R package installed from Bioconductor, which can be used to explore functional profiles of genes or gene clusters [13]. GO annotations explain the function of genes from three aspects: possible molecular functions, cellular environment, and biological processes. KEGG is a bioinformatics repository that contains comprehensive information related to biological pathways. Enrichment analysis results with statistical significance (*p* value < 0.05) were screened out and visualized by bar graph and bubble map.

### 2.5. Gene Set Enrichment Analysis

By analyzing whole gene expression profile data, Gene Set Enrichment Analysis (GSEA) determines whether there are statistical differences in the expression of specific gene sets in different biological phenotypes [14]. This algorithm includes genes that are not significantly differentially expressed but are biologically important and is complementary to GO and KEGG analysis. Hallmark gene sets from Molecular Signatures Database (MSigDB) were chosen as reference gene sets [15]. The results meeting following thresholds were significant: |normalized enrichment score (NES)| > 1, nominal *p* value < 0.05, and FDR *q* value < 0.25.

### 2.6. Protein-Protein Interaction Network

In order to clearly present the interaction network between proteins encoded by DEGs and find out hub genes, we constructed protein-protein interaction (PPI) network. First, we imported the list of DEGs into STRING database [16], filtered the network with medium confidence value (0.4), and hid isolated nodes. Next, Cytoscape software was selected for detailed processing and visual analysis. The Molecular Complex Detection (MCODE) plug-in in Cytoscape can detect closely related nodes in a large network and classify them into different clusters. Genes in the highest scoring cluster were considered as hub genes.

### 2.7. Prediction of Pivotal MicroRNAs and Transcription Factors

Functional Enrichment analysis tool (FunRich) was used to predict microRNAs (miRNAs) targeting hub genes. The transcription factors (TFs) were predicted using Database for Annotation, Visualization and Integrated Discovery (DAVID) [17]. A threshold of *p* value < 0.05 was used for filtering analysis results. Moreover, gene-miRNA interaction network and gene-TF interaction network were further processed in Cytoscape.

### 2.8. Validation of Hub Genes

We downloaded the gene expression profiling data of GSE60993, including 7 ACS patients and 7 healthy controls [18]. The expression values of hub genes in the matrix were used to draw receiver operating characteristic (ROC) curves and calculate area under curve (AUC) by pROC package [19]. AUC value reflects the sensitivity and specificity of a gene in distinguishing ACS from health. Here, we believed that genes with AUC greater than 0.9 can be used as biomarkers for the diagnosis of ACS.

## 3. Results

### 3.1. DEG Screening

**Figure 1:**
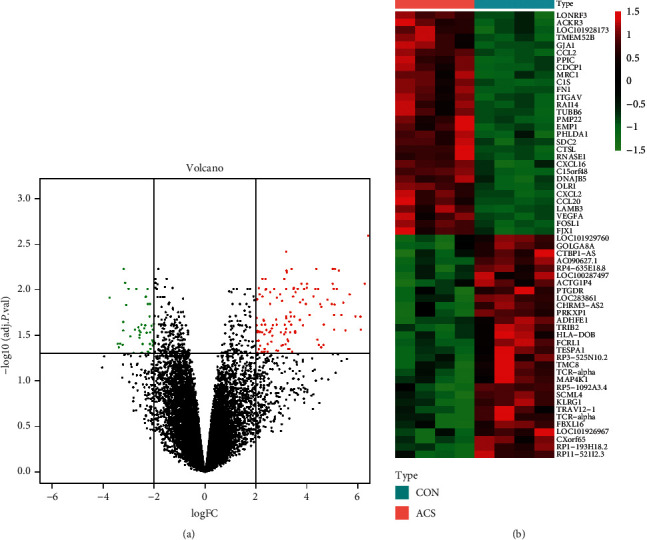
(a) The volcano plot was presented, in which green dots represented downregulated genes and red dots represented upregulated genes in ACS samples. (b) Heat map of gene expression. Each row represented one DEG, and the color gradually changed from green to red, indicating the shift of gene expression from low to high. ACS: acute coronary syndrome; DEG: differentially expressed gene.

A total of 176 DEGs between ACS and control were finally screened out according to the above criteria, including 130 upregulated genes and 46 downregulated genes. The DEGs were visualized by volcano plot and heat map, as shown in Figures 1(a) and 1(b).

### 3.2. Functional Enrichment of DEGs

**Figure 2:**
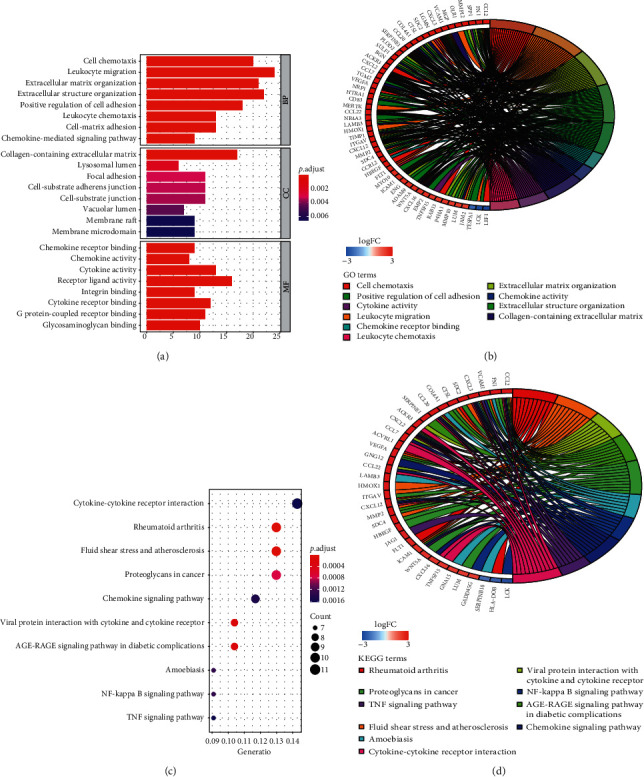
(a) Top 8 items of BP, CC, and MF shown in the bar chart according to adjust *p* value. (b) GO chord plot and the order of genes arranged by logFC. (c, d) KEGG pathways enriched by DEGs presented by bubble plot and chord plot, respectively. BP, biological process; CC, cellular component; MF, molecular function; GO, Gene Ontology; logFC, log2 fold change; KEGG, Kyoto Encyclopedia of Genes and Genomes.

Gene function annotations of DEGs were primarily enriched in activation and migration of inflammatory cells and immune response. The top eight BP, CC, and MF terms are shown in Figure 2(a), according to the order of adjusted *p* value. In addition, we used GOplot R package to draw a circle diagram, which clearly presented the correspondence between GO terms and genes (Figure 2(b)). Similarly, top 10 KEGG pathways are shown in Figures 2(c) and 2(d). The results suggested that DEGs were mainly involved in immune-related pathways, such as cytokine-cytokine receptor interaction, NF-*κ*B signaling, and atherosclerosis.

### 3.3. Gene Set Enrichment Analysis

**Figure 3:**
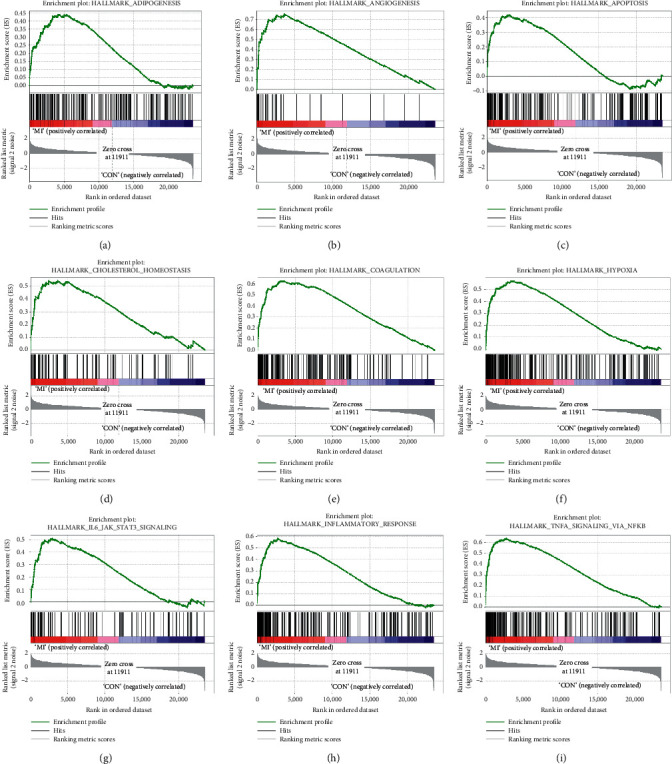
Gene set enrichment analysis of GSE19339. Significantly enriched gene sets were selected based on threshold values: | normalized enrichment score (NES)| > 1, nominal *p* value < 0.05, and FDR *q* value < 0.25.

Gene expression matrix and phenotype file were prepared to uncover gene sets that were significantly enriched in ACS group. Based on hallmark gene set database, we found that some of enrichment results were similar to GO and KEGG analysis, such as inflammatory response and TNF-*α* signaling via NF-*κ*B. Additionally, enrichment outcomes including coagulation, cholesterol homeostasis, hypoxia, and apoptosis were also closely related to the pathophysiological process of ACS (Figure 3).

### 3.4. PPI Network Construction and Gene Cluster Identification

**Figure 4:**
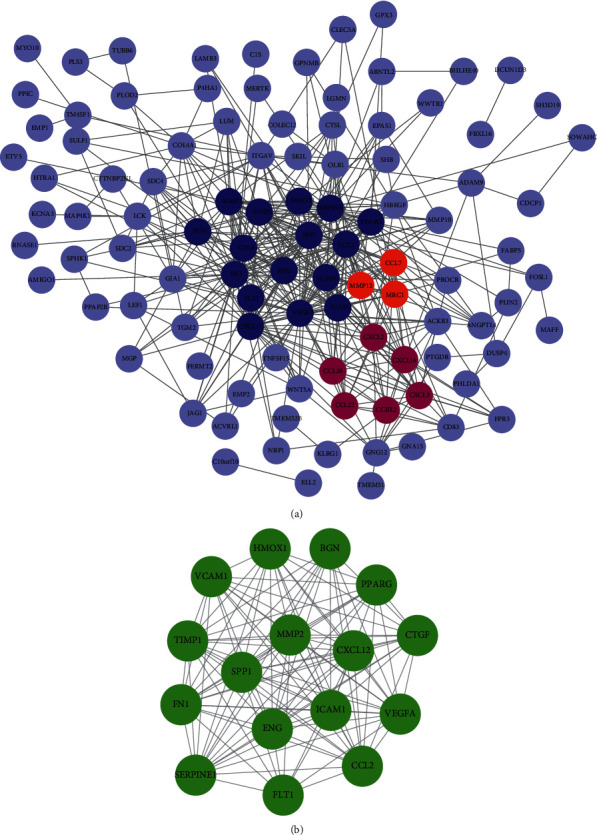
(a) The PPI network analysis for DEGs. Different clusters identified by MCODE were marked with different colors. Sixteen dark blue dots were cluster 1, six purple dots were cluster 2, and three orange dots were cluster 3. (b) The PPI network of 16 hub genes. PPI: protein-protein interaction; MCODE: molecular complex detection.

After filtering original network with medium confidence value and hiding isolated nodes, the network was transferred to Cytoscape for detailed processing. As shown in Figure 4(a), the PPI network consisted of 103 nodes plus 440 edges. The MCODE plug-in identified a total of three gene clusters, among which cluster 1 having 16 nodes and 111 edges scored the highest (Figure 4(b)). Genes in this cluster were thought to be central to the development of ACS, so we uploaded them to DAVID database for enrichment analysis to further clarify their biological effects. The results suggested that hub genes were primarily involved in angiogenesis and inflammation-related functions (Table 1).

### 3.5. Further MiRNAs and TF Mining

**Figure 5:**
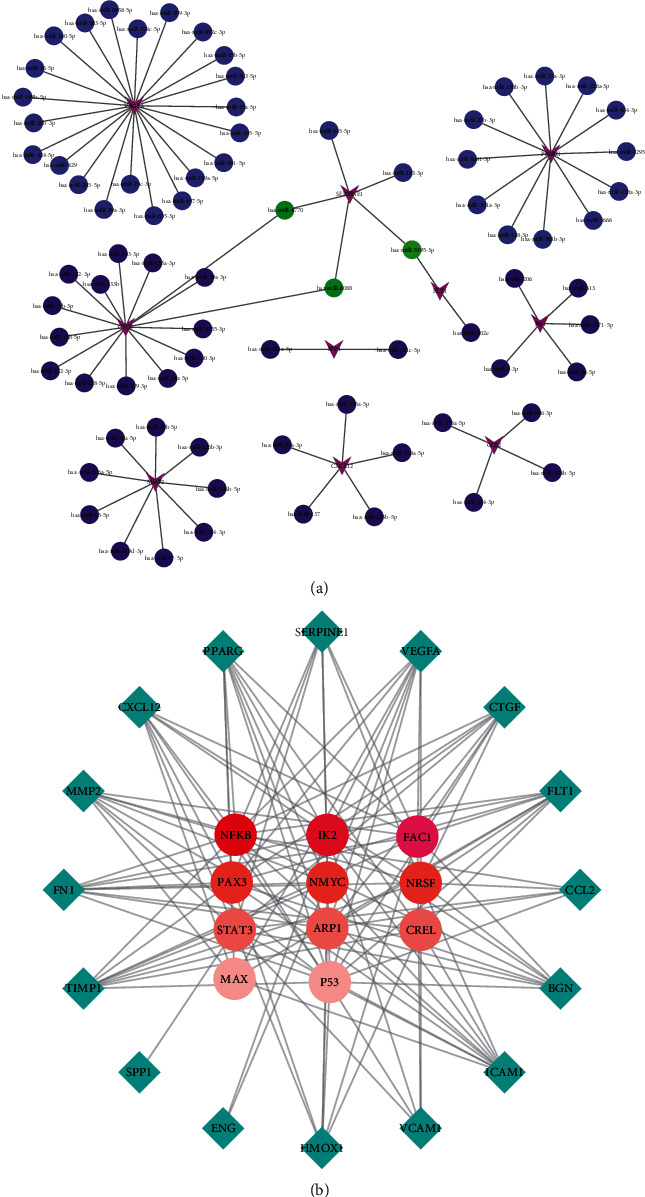
(a) Interaction network between miRNAs and their target genes. Genes were indicated by purple arrows and miRNAs were represented by blue circles. Moreover, miRNAs targeting two genes were shown by green circles. (b) Interaction network between genes and TFs. The diamonds represented genes and circles indicated TFs. The smaller the *p* value, the darker the circle color. miRNAs: microRNAs; TFs: transcription factors.

Sixteen hub genes in the highest scoring cluster were uploaded to FunRich software for miRNAs analysis. Among the predicted results, miRNAs possessing three target genes, including hsa-miR-4770, hsa-miR-5195, and hsa-miR-6088, were considered to be key regulators of the pathological process of ACS. Subsequently, we used Cytoscape to visualize the regulatory network between miRNAs and hub genes, as shown in Figure 5(a). With the help of DAVID database, we searched for proteins that regulate hub gene transcriptional processes, known as TFs. A total of 11 transcription factors were identified, and the top three ranked by *p* value were NF-*κ*B, IK2, and FAC1. The interaction network between hub genes and TFs is shown in Figure 5(b).

### 3.6. ROC Curve Verification

**Figure 6:**
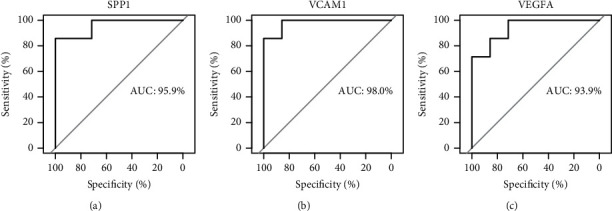
ROC curves of three hub genes with excellent diagnostic value for ACS. The AUC was calculated for each plot. ROC: receiver operating characteristic; AUC: area under curve.

Validated by ROC curves, we found that 3 of the 16 hub genes had high sensitivity and specificity, including VEGFA (AUC = 0.939), SPP1 (AUC = 0.959), and VCAM1 (AUC = 0.98) (Figure 6). The three genes may be biomarkers of ACS and have positive implications for early medical intervention of the disease.

## 4. Discussion

ACS is a disease with high morbidity that seriously threatens life quality and survival time of patients. In this study, we first screened out 176 DEGs, including 130 upregulated genes and 46 downregulated genes. Then, databases including GO and KEGG were selected to do gene enrichment analysis, and the results suggested that these genes were primarily involved in inflammatory response signaling. From the results of GSEA, it could be seen that coagulation, cholesterol homeostasis, hypoxia, and apoptosis also played a key role in the pathogenesis of ACS. In order to find hub genes, PPI network of DEGs was set up in Cytoscape, and MCODE plug-in analysis was performed. The highest scoring cluster contains 16 hub genes, which were CXCL12, FN1, CTGF, BGN, ENG, HMOX1, VEGFA, CCL2, FLT1, SPP1, VCAM1, PPARG, TIMP1, MMP2, SERPINE1, and ICAM1. Hub genes related miRNAs and TFs were further mined by FunRich software and DAVID database, respectively. Furthermore, another microarray dataset (GSE60993) was selected to plot ROC curves to assess the sensitivity and specificity of hub genes in ACS diagnosis. Three genes with AUC > 0.9, including VEGFA, SPP1, and VCAM1, had excellent reliability as indicators for disease prediction and early intervention.

Chemokines play a guiding role in leukocyte migration to the inflammatory site by binding to *G* protein coupled receptors [20]. Cytokines are small proteins secreted by immune cells and some nonimmune cells that have a wide range of biological activities. They bind to receptors on the surface of cell membrane, activate intracellular signal transduction pathways, and play an important role in maintaining homeostasis in the body. In the process of atherosclerotic plaque formation, lipid particles are trapped in the arterial wall [21] and endothelial cells express adhesion molecules in response to modified lipoproteins [22]. Then circulating immune cells are recruited to these sites and produce proinflammatory mediators such as tumor necrosis factor (TNF), which elicits local inflammation [23, 24]. In addition, infiltrating monocytes differentiate into macrophages, and sustained phagocytosis of lipoproteins converts them into foam cells, a major component of atherosclerotic plaques.

MiRNAs are noncoding RNAs, which have the function of negatively regulating gene expression at translational level [25]. MiR-6088, identified in 2012, was found to be differentially expressed during endothelial cell differentiation [26]. Another study showed that miR-6088 was closely associated with tumor cell proliferation and migration [27]. MiR-5195, which was found in deep sequencing of small RNA in acute lymphocytic leukemia, was involved in tumor cell invasion and metastasis [28, 29]. Similarly, the expression level of miR-4770 was linked to breast cancer [30]. Although the role of three key miRNAs in regulation of ACS has not been reported, they may influence atherosclerotic plaque progression by promoting cell proliferation and transformation. Transcription factors are proteins that regulate gene expression by binding with corresponding DNA sequence to enhance or block the recruitment of target genes to RNA polymerase. In our study, we predicted 11 TFs regulating hub genes via DAVID database. NF-*κ*B, the most significant transcription factor in analysis results, which promoted the expression of several proinflammatory genes such as VCAM1 in endothelial cells, increased macrophage recruitment [31, 32].

Extensive studies have confirmed the potential value of three hub genes in the diagnosis of ACS. SPP1, also known as OPN, is a calcium-binding glycosylated phosphoprotein associated with bone formation, inflammation, and vascular calcification [33]. Its expression would upregulate when calcium was deposited in atherosclerotic plaques [34]. A study of 120 subjects conducted in 2000 first proposed that there was a positive relationship between SPP1 and coronary artery disease, and SPP1 might be a potential biomarker to identify patients with or at risk for ACS [35]. Another study confirmed that SPP1, as a hub gene, was significantly over expressed in patients with carotid plaque rupture, suggesting that it was involved in plaque instability and had a predictive role in plaque rupture [36]. In atherosclerosis model mice, knockdown of SPP1 not only reduced atherosclerotic lesion size but also decreased the number of macrophages in the plaque. Thus, SPP1 could slow disease progression via regulating the number of immune cells and suppressing inflammatory response [37]. Vascular endothelial growth factor (VEGF), which can be subdivided into VEGFA, VEGFB, and VEGFC, is a substance that increases vascular permeability and promotes endothelial cell migration and proliferation [38]. VEGFA, an important component of the VEGF family, is an indispensable growth factor for intraplaque angiogenesis and is directly related to plaque stability. Under hypoxic conditions, VEGFA bound to its receptor, activating mitogen-activated protein kinase (MAPK), which induced endothelial cell proliferation, macrophage infiltration, and foam cell formation [39]. VCAM1, a member of immunoglobulin superfamily, was combined with integrins VLA-4, favoring the recruitment of leukocytes and thus aggravating atherosclerotic plaque [40]. All in all, known biological roles of the three hub genes strengthen the reliability of our results.

Some limitations in the study should be noted. Firstly, dataset GSE19339 selected in our study covered a small sample size and was not jointly analyzed with other datasets. The reason was that we wanted to explore hub genes closely related to the occurrence of plaque rupture. Therefore, only studies with thrombus as specimen were included. Secondly, the results were not experimentally validated. To compensate for this limitation, another dataset (GSE60993) from GEO database was chosen to plot ROC curves and calculate corresponding AUC values, which helped to improve the reliability of our findings. In the following work, relevant clinical and animal experiments will be conducted to explore the molecular mechanism of hub genes in ACS and provide potential targets for disease intervention.

## 5. Conclusion

Our study reveals three diagnostic markers of ACS, including VCAM1, SPP1, and VEGFA, which may influence disease progression by mediating immune responses and inflammation-related pathways.

## Figures and Tables

**Table 1:** Biological processes associated with hub genes in cluster 1.

Biological process	Gene count	FDR
Angiogenesis	8	5.60*E *−* *07
Response to hypoxia	7	7.05*E *−* *06
Cell adhesion	8	8.33*E *−* *05
Extracellular matrix disassembly	5	7.32*E *−* *04
Extracellular matrix organization	6	8.08*E *−* *04
Positive regulation of angiogenesis	5	0.003863389
Cell chemotaxis	4	0.035011825

## Data Availability

The data that support the findings of this study are openly available.
